# Schistosomiasis Control in Ethiopia: The Role of Snail Mapping in Endemic Communities

**DOI:** 10.3390/tropicalmed7100272

**Published:** 2022-09-28

**Authors:** Asrat Meleko, Sarah Li, Dorin Brener Turgeman, Michal Bruck, Nisan Z. Kesete, Willemijn Zaadnoordijk, David Rollinson, Galia Sabar, Zvi Bentwich, Rachel Golan

**Affiliations:** 1 NALA, Carlebach 29, Tel Aviv-Yafo 6713224, Israel; 2 Department of Public Health, Mizan Tepi University College of Medicine and Health Sciences, Tepi 5160, Ethiopia; 3 Department of Population Health, New York University, New York, NY 10012, USA; 4 Ares Trading S.A., 1262 Eysins, Switzerland; 5 Department of Life Sciences, The Natural History Museum, London SW7 5BD, UK; 6 Global Schistosomiasis Alliance, London W5 2RJ, UK; 7 The Department of Middle Eastern and African History, Tel Aviv University, Tel Aviv-Yafo P.O. Box 39040, Israel; 8 Shraga Segal Department of Microbiology, Immunology and Genetics, Ben-Gurion University of the Negev, Beer Sheva P.O. Box 653, Israel; 9 Department of Epidemiology, Biostatistics and Community Health Sciences, Ben-Gurion University of the Negev, Beer Sheva P.O. Box 653, Israel

**Keywords:** schistosomiasis, snail mapping, cercariae, neglected tropical diseases, Ethiopia

## Abstract

Introduction: Schistosomiasis, a neglected tropical disease (NTD), remains a public health problem in Ethiopia. Freshwater snails, acting as intermediate hosts, release cercariae, the infectious parasite, into the water, which penetrate human skin that encounters infested waters. The objective of this study was to map snail abundance along rivers and study its association with schistosomiasis infection in communities using these rivers. Materials and Methods: A cross-sectional study was carried out at 20 river sites in Mizan Aman city administration, Bench Sheko zone, South West Ethiopia Peoples (SWEP) region, Ethiopia, to study the distribution of host snails and transmission sites for intestinal schistosomiasis. This study used a quantitative database consisting of data on the prevalence of infected snails, the characteristics of rivers and riverbanks, and the prevalence of schistosomiasis in the community, based on stool samples collected from community members near the sampling sites. Results: Aquatic snails were found in 11 of the 20 sites sampled. A total of 598 snails was collected, including *Biomphalaria pfeifferi*, *Biomphalaria sudanica*, *Radix natalensis* and *Bulinus globosus* species; the most abundant species was *Biomphalaria pfeifferi.* Stool samples were collected from 206 community members from all 20 sites. Forty-one (19.9%) were positive for *Schistosoma mansoni*. A positive correlation was found between the presence of snails and positive stool samples (r = 0.60, *p* = 0.05) and between the presence of infected snails and the prevalence of infection (r = 0.64, *p* = 0.03). Locations with muddy riverbanks were associated with the presence of snails (r = 0.81, *p* < 0.001). Conclusions: These results emphasize the importance of mapping snails for the control of schistosomiasis by defining hotspots of infection and identifying factors associated with the presence of infected snails. The results support the need for a continuous mapping of snails and the introduction of snail control as a major element for the successful control of schistosomiasis in endemic communities.

## 1. Introduction

Schistosomiasis, a neglected tropical diseases (NTD), is caused by blood flukes (termotode worms) called *Schistosoma.* [1]. Although the disease is both treatable and preventable, it remains a consistent problem in tropical and subtropical regions of several low- and middle-income countries, with 90% of cases found in sub-Saharan Africa. In 2020, the Ethiopian Federal Ministry of Health (FMoH) mapped 346 schistosomiasis endemic districts in Ethiopia [2]. Associated with a lack of access to clean water and sanitation [3], this equates to approximately 5 million people infected with schistosomiasis and 37.5 million people at an increased risk of infection in Ethiopia [4].

Schistosomiasis is transmitted via the intermediate snail host through the release of ova from human infected feces or urine, dependent on the schistosome species [5]. The main species found in Ethiopia are *Schistosoma haematobium (S. haematobium)*, the cause of urogenital schistosomiasis, and *Schistosoma mansoni (S. mansoni)*, the cause of intestinal schistosomiasis. In water, the eggs hatch and release miracidia that infect freshwater snails, the schistosome intermediate host. Different species of *Schistosoma* target different species of snails, primarily *Bulinus* spp. for *S. haematobium* and *Biomphalaria* spp. for *S. mansoni.* Cercariae released from infected snails can then penetrate the human skin in contact with infested waters. Inside the human host, cercariae mature into egg-laying worms and migrate to their preferred egg-laying site. Domestic, occupational, and recreational activities near rivers increase the likelihood of contamination due to human excrement, which results in parasite eggs being released into the water, infecting snails, and continuing the life cycle of the parasite [6]. Therefore, one component of effective disease prevention is the mapping, surveillance, and control of the snail population [5].

Utilizing snail (vector) control as a method for reducing snail-transmitted parasitic infections includes diverse and sometimes controversial measures, including, but not limited to, applying molluscicides and bioremediation to eliminate snail populations [7], plant control measures to make habitats unsuitable for snail proliferation [1], or biocontrol methods such as introducing competitor snails [8]. Other, less environmentally altering measures include community behavioral change programs [7] and investment in improving access to, and practices of, Water, Sanitation and Hygiene (WASH). However, for all of these measures, snail mapping is crucial for identifying the distribution of snail populations, the prevalence of snails infected with schistosomiasis, and high-transmission areas [9].

In this study, a snail mapping survey was conducted to examine the association between snail abundance, cercariae, community interaction, and prevalence of schistosomiasis in Mizan Aman city administration, Bench Sheko zone, SWEP region, Ethiopia.

## 2. Methods

A cross-sectional study was conducted between October and December 2021, in Mizan Aman city administration, Bench Sheko zone, SWEP region, Ethiopia, to examine the distribution of host snails and potential transmission sites for intestinal schistosomiasis and their association with disease prevalence in the community.

Twenty sampling sites were selected for the study based on the following criteria: rivers frequently used by the community; proximity of the rivers to houses; rivers located in communities with registered human cases of schistosomiasis; and rivers with easy road access. The identified sites were geo-coordinated and mapped using the Global Positioning System (GPS). The geographical coordinates of the snail sites were recorded on a hand-held Geographical Positioning System (Garmin, Olathe, KS, USA).

Five technicians conducted the snail collection and environmental measurements between 8 am and 11 am at each site. The snails were collected by hand in areas with shallow bodies of water and with dip scoops in areas with large bodies of water, for 20–30 min. Rain boots and hand gloves were used during sampling. The snails collected were identified by species, labeled, and transported to the SWEP regional parasitological laboratory in perforated plastic containers for analysis. Snail species were identified based on shell morphology using Mandahl-Barth’s identification key for ‘East and Central African snails of medical and veterinary importance’ [10]. The snails were then tested for natural trematode cercariae infections using the shedding method. Each snail was individually placed in shedding vials with approximately 100 mL of deionized water and then exposed to an electric light for approximately three hours. The vials that showed no shedding of cercariae after the first exposure were re-exposed to the electric light on subsequent days, for a maximum of five day. The tail morphology of the cercariae shed was used for genus identification. The number and type of vector snails and trematode cercariae identified were recorded. The precise identification of the snails using molecular methods was not possible; therefore, the taxonomic designations await confirmation.

Environmental and physicochemical parameters of the rivers were examined at all sites, including pH, temperature, salinity, total dissolved solids, and conductivity of the water. A photometer was used to measure total dissolved solids, a pH-meter for pH, and a probe and meter were used to measure water conductivity. The environmental data included parameters relating to vegetation, riverbank characteristics, and presence of animals.

Stool samples were collected from young community members (age range, 10–29 years) living near the sampling sites, to examine the prevalence of infection among the community. Written consent was obtained from the parents of the participants under the age of 18. Community members were asked whether they used these rivers for their livelihood and if they had alternatives, as well as whether they were aware of the association between snails in the river and diseases. The Ethical Review Committee of Mizan Tepi University College of Medicine and Health Sciences provided ethical approval for this study. In addition, the Bench Sheko zonal health department and the Mizan Aman city administration provided letters of support and permission. Written consent was obtained from local residents who provided stool samples.

### Statistical Analysis

Descriptive statistics were used to describe the sampling sites, riverbanks, snails, and local population. Chi squared test and Spearman correlation were used to examine associations between snail abundance, snail infectivity, substratum, physicochemical variables, vegetation types, human activities, and presence of schistosomiasis in the stool samples. The EpiData Version 4.4.2.1 software (EpiData Association, Odense, Denmark) was used to enter, code, and clean the data. SPSS version 25.0 was used to analyze the data (IBM Corp. IBM SPSS Statistics for Windows, Armonk, NY, USA). The distribution analysis was carried out using the ArcGIS system for the Geographical Information System (GIS) for proximity analysis. A *p*-value of < 0.05 was considered statistically significant.

## 3. Results

A total of 20 snail collection sites (rivers) used regularly by the community were included. Aquatic snails were found in 11 of the 20 sites sampled (55.0%). In the remaining nie sites, no snails were detected. A total of 598 snails were collected. Four snail species were found: *Biomphalaria pfeifferi*, *Biomphalaria sudanica*, *Radix natalensis*, and *Bulinus globosus*. Of the total snails collected, 59.9% were *B. pfeifferi*, 24.6% *B. sudanica*, 15.0% *Radix natalensis*, and 0.5% *Bulinus globosus* (Table 1). Nine of the eleven (82%) collection sites had snails that tested positive for cercariae. The results from cercariae shedding showed that 13.0% of the total snails collected were infected. Thirteen percent of *Biomphalaria* snails were found to be infected. Specifically, of 358 *B. pfeifferi* snails collected, 36 (10.1%) shed cercariae, whilst 30 (20.4%) of the 147 *B. sudanica* shed cercariae. Of the 90 *L. natalensis* snails collected, 13 (13.3%) shed non-schistosome cercariae, while none of the *Bulinus globosus* were found to be shedding cercariae (Table 1). The mean water temperature was 24.7 °C ± 2.7 (range = 18.5 °C to 29.0 °C). The water pH levels varied between sites, with a mean pH of 7.3 ± 0.7 (range = 5.3−8.5). The mean water conductivity was 120.2 ± 110.8 mS/cm (range: 50.50–551.00 mS/cm), and the mean water salinity was 58.3 ± 54.1 (range: 32.0–700.0) (Table 2).

A significant association between total snail abundance and salinity was found (r = 0.76, *p* = 0.007). The presence of mud along riverbanks was found to be associated with the presence of snails (r = 0.81, *p* < 0.001), while the presence of sand, gravel, or rocks was not associated with snail presence. Water temperature (r = −0.16, *p* = 0.65), water pH (r = 0.06, *p* = 0.87), and water conductivity (r = −0.3, *p* = 0.32) were not associated with snail presence in this study. Water depth was associated with the number of snails found (r = 0.65, *p* = 0.03), and water level was associated with the number of infected snails collected (r = 0.69, *p* = 0.02). Since the study was conducted during the dry season, most rivers had low water levels and flowed slowly.

Various types of vegetation were found, including Taro (Godare), banana trees and grasses as the most common vegetation observed in all habitats. Other vegetation types present in some of the selected sites included sugarcane and inset (false banana). A Large Taro (Godare) vegetation cover was observed in sites with a high snail abundance. Both *Biomphalaria pfeifferi* and *B. sudanica* were found in rivers with a variety of vegetation, but they were most common in areas with a high population of Taro (Godare) and water grass. Open defecation was observed near riverbanks, in 9 of the 20 sites.

Stool samples were collected from a total of 206 participants (48% girls) from all 20 sampling sites (Table 3). Positive stool samples for schistosomiasis were found in 41 (19.9%) samples. A positive correlation was determined between the number of snails found and collected in the rivers (11 out of 20 sites) and the prevalence of infection (as detected by positive stool samples) (r = 0.60, *p* = 0.05). Furthermore, a positive correlation was found between the number of infected snails collected and prevalence of infection (r = 0.64, *p* = 0.03).

Despite the presence of infected *Biomphalaria* snails in the rivers sampled, the community relies heavily on these rivers for their daily activities. The participants reported gaps in awareness of the communities with regard to the relationship between the water, the infected snails, and the disease.

## 4. Discussion

This cross-sectional study examined the distribution of host snails and transmission sites for intestinal schistosomiasis and its association with infection in the community at 20 river sites in Mizan Aman city administration. The most abundant snail species found was *Biomphalaria pfeifferi,* followed by *Biomphalaria sudanica*, *Radix natalensis*, and *Bulinus globosus.* Significant associations were found between total snail abundance and salinity, muddy riverbanks, and water depth. Positive correlations were found between snail abundance and number of infected individuals and between infected snails and infected individuals. These findings demonstrate that the majority of the rivers examined were a suitable habitat for *Biomphalaria* species, the primary intermediate host of *S. mansoni.* The precise identification of the *Biomphalaria* species involved in transmission and the schistosome cercariae observed in these habitats will require more detailed molecular characterization to complement the morphological identifications.

The sites were chosen from preliminary observations of high levels of human-to-water contact, and this study shows how various parameters correlated with snail abundance and, subsequently, infection with cercariae. However, this preliminary study was conducted between October and December, and the parameters vary seasonally. Associations were found between snail abundance and water salinity, as well as between snail abundance and muddy substrate on riverbanks. Yet, no correlation was found between snail abundance and water temperature. These data conflict with other studies that show a correlation between these two variables, as cooler temperatures result in snails proliferating at a slower rate [1,10]. Yigezu G. et al. found correlations between other physicochemical properties, such as water conductivity, pH, turbidity, hardness, and total dissolved oxygen, and snail abundance [1]. This suggests that the relative importance of specific environmental determinants and their effects on snail abundance cannot be generalized. This claim is supported by Salew D. et al., who showed that there is no one key environmental determinant that contributes to snail abundance [11]. While the water physicochemical properties have little impact on snail abundance, the most important factor may be the level of human activity [1,12,13].

The control of schistosomiasis is mainly conducted by treating the disease through mass drug administration (MDA) programs [2]. However, MDAs do not prevent reinfection. Thus, an integrated approach to schistosomiasis prevention and control should be considered for the long-term elimination of the disease, complementing MDAs with the maintenance and implementation of WASH facilities and snail mapping. The WHO is reinforcing snail control as part of its strategic approach to eliminating schistosomiasis as a public health problem and, ultimately, to achieving a total break in transmission [9]. Snail control should therefore supplement MDA campaigns as an opportunity for maintaining a low prevalence of the disease.

Implementing environmentally friendly snail control methods would aid in the long-term elimination of schistosomiasis. All infected sites were close to, or on the way to, schools, requiring students to cross them. Community-wide interventions include signage prohibiting students from playing or swimming in the river or zoning the safe water, building a bridge for safe crossing, educating the community regarding the potential risk factors associated with a constant contact with contaminated rivers, and strengthening health messages in order to avoid open defecation near rivers. An additional solution could include removing vegetation and covering muddy substrate with gravel or sand, in accordance with several studies that have reported the importance of certain vegetation in snail proliferation [1,6,14,15,16].

The community participants reported to the study technicians that they did not have alternative water sources and were unaware of the association between aquatic snails present in the water and schistosomiasis. Although this study did not practice citizen science per se [17], a preliminary study conducted by Brees et al. reported that training local citizens in collecting, identifying, and mapping snails was an effective way of generating a large amount of data to be used for local targeted snail control measures [18]. Citizen science as a means of collecting more data on snail abundance and species identification is a low-cost and efficient method of snail mapping and subsequent snail control and should be considered along with practicing behavioral changes with the community regarding the potential risks associated with a constant contact with contaminated rivers. The cultural and utility importance of these water sources in the daily lives of those who rely on them must also be considered.

Since the study was conducted during the dry season, it is important to repeat it during the rainy season in order to evaluate additional factors that may influence snail distribution. A study conducted by Clennon in 2006 [6] reported that during the wet season, snail elimination methods proved ineffective, suggesting that the increasing water levels connected different pools, creating corridors for migration and thus facilitating the movement of snails from one pool to another.

## 5. Conclusions

This study emphasizes the importance of mapping snails for the control of schistosomiasis by defining hotspots of infection and identifying factors associated with the presence of infected snails. To achieve the long-term elimination of schistosomiasis, it is necessary to provide public health education focusing on water, sanitation, hygiene, and the schistosome life cycle and route of transmission, as well as community-wide interventions, alongside MDA campaigns, in order to reduce disease transmission and re-infection in endemic communities.

## Figures and Tables

**Table 1:** Characteristics of the collected snails across the sampling sites n = 598.

Sampling Site	*Biomphlaria pfeifferi*	*Biomphlaria sudanica*	*Lamina natalensis*	*Bulinus glubuses*	# Infected Snails/# Snails [%]
Agu 1	1/48 [2.1]	3/22 [13.6]	0/8 [0.0]	0/0 [0.0]	4/78 [5.1]
Agu 2	9/36 [25.0]	9/18 [50.0]	0/6 [0.0]	0/0 [0.0]	18/60 [30.0]
Sasin	5/101 [4.9]	5/37 [13.5]	4/30 [13.3]	0/1 [0.0]	14/169 [6.3]
Shonga 1	0/54 [0.0]	5/29 [17.2]	6/41 [14.6]	0/0 [0.0]	11/124 [8.9]
Shonga 2	0/1 [0.0]	0/0 [0.0]	0/0 [0.0]	0/0 [0.0]	0/1 [0.0]
Shonga 3	0/0 [0.0]	0/0 [0.0]	0/0 [0.0]	0/0 [0.0]	0/0 [0.0]
Kusha	17/56 [30.4]	3/15 [20.0]	0/0 [0.0]	0/0 [0.0]	20/71 [28.2]
Keker 1	0/0 [0.0]	0/0 [0.0]	0/0 [0.0]	0/0 [0.0]	0/0 [0.0]
Keker 2	0/0 [0.0]	0/0 [0.0]	0/0 [0.0]	0/0 [0.0]	0/0 [0.0]
Esine 1	1/12 [8.3]	0/0 [0.0]	2/5 [40.0]	0/0 [0.0]	3/17 [17.6]
Esine 2	0/0 [0.0]	0/0 [0.0]	0/0 [0.0]	0/0 [0.0]	0/0 [0.0]
Esine 3	0/0 [0.0]	0/0 [0.0]	0/0 [0.0]	0/0 [0.0]	0/0 [0.0]
Kosokol 1	1/40 [2.5]	1/20 [5.0]	0/0 [0.0]	0/1 [0.0]	2/61 [3.3]
Kosokol 2	1/1 [100.0]	0/0 [0.0]	0/0 [0.0]	0/0 [0.0]	1/1 [100.0]
Kosokol 3	0/0 [0.0]	0/0 [0.0]	0/0 [0.0]	0/0 [0.0]	0/0 [0.0]
Kosokol 4	0/0 [0.0]	0/0 [0.0]	0/0 [0.0]	0/0 [0.0]	0/0 [0.0]
Chorobay	1/7 [14.3]	4/6 [66.7]	0/0 [0.0]	0/1 [0.0]	5/14 [35.7]
Borini	0/0 [0.0]	0/0 [0.0]	0/0 [0.0]	0/0 [0.0]	0/0 [0.0]
Kabash	0/0 [0.0]	0/0 [0.0]	0/0 [0.0]	0/0 [0.0]	0/0 [0.0]
Wugni	0/2 [0.0]	0/0 [0.0]	0/0 [0.0]	0/0 [0.0]	0/2 [0.0]
Total	36/358 [10.1]	30/147 [20.4]	12/90 [13.3]	0/3 [0.0]	78/598 [13.0]

**Table 2:** Characteristics of the rivers across the sampling sites n = 20.

Sampling Site	Temperature (°C)	pH	Salinity (g/I)	Total Dissolved Solids (mg/I)	Conductivity (mS)
Agu 1	23.9	7.8	210	53.3	106.8
Agu 2	20	6.9	110	64.9	130.2
Sasin	26	6.9	350	42	68.8
Shonga 1	26.2	6.6	450	29.2	58.5
Shonga 2	25.4	6.8	90	53	85
Shonga 3	26	7.4	700	25.2	50.5
Kusha	26	7.1	125	63.2	60
Keker 1	25.8	8.1	95	30	62.6
Keker 2	25.1	7.4	200	31.4	64
Esine 1	20	7.4	100	36	73
Esine 2	22.9	7.4	85	35.8	85.9
Esine 3	26.1	7.5	86	57.9	115.8
Kosokol 1	28	8.5	99	81.3	162.7
Kosokol 2	26.5	7.2	100	57.3	114.9
Kosokol 3	23	7.2	32	22	97
Kosokol 4	18.5	7.2	50	76.5	153
Chorobay	26.1	5.3	50	31.7	63.5
Borini	26.3	7.6	150	70	231
Kabash	22.5	8.1	550	275	551
Wugni	29	7.3	113	30	70
Mean ± SD	24.7 ± 2.7	7.3 ± 0.6	187.3 ± 182.2	58.3 ± 54.1	120.2 ± 110.83

**Table 3:** Characteristics of the study population across the sampling sites n = 206.

Sampling Site	Sex (# Cases/n), [%]		Age, Years	Total # Schistosomiasis Cases
	Female	Male		
Agu 1	1/9 [11.1]	3/6 [50.0]	15.6 ± 3.6	4/15 [26.7]
Agu 2	3/8 [37.5]	1/5 [20.0]	14.5 ± 3.0	4/13 [30.8]
Sasin	1/5 [20.0]	2/5 [40.0]	15.6 ± 1.6	3/10 [30.0]
Shonga 1	1/5 [20.0]	3/12 [25.0]	14.5 ± 1.7	4/17 [23.5]
Shonga 2	0/0 [0.0]	0/4 [0.0]	13.5 ± 2.4	0/4 [0.0]
Shonga 3	4 [0.0]	5 [0.0]	19.9 ± 3.2	0/9 [0.0]
Kusha	1/5 [20.0]	3/10 [30.0]	15.3 ± 3.1	4/15 [26.7]
Keker 1	1/8 [12.5]	1/6 [16.7]	12.9 ± 2.3	2/14 [14.3]
Keker 2	0/8 [0.0]	2/10 [20.0]	14.7 ± 2.0	2/18 [11.1]
Esine 1	6/10 [60.0]	0/7 [0.0]	16.0 ± 5.3	6/17 [35.3]
Esine 2	0/7 [0.0]	1/1 [100.0]	12.9 ± 2.2	1/8 [12.5]
Esine 3	0/2 [0.0]	0/4 [0.0]	20.2 ± 5.3	0/6 [0.0]
Kosokol 1	1/5 [20.0]	2/5 [40.0]	19.8 ± 5.2	3/10 [30.0]
Kosokol 2	0/3 [0.0]	0/3 [0.0]	18.0 ± 2.0	0/6 [0.0]
Kosokol 3	0/4 [0.0]	0/3 [0.0]	14.6 ± 2.2	0/7 [0.0]
Kosokol 4	1/3 [33.3]	0/3 [0.0]	17.0 ± 5.3	1/6 [16.7]
Chorobay	2/4 [50.0]	1/4 [25.0]	15.2 ± 3.7	3/8 [37.5]
Borini	0/2 [0.0]	0/3 [0.0]	16.8 ± 5.9	0/5 [0.0]
Kabash	2/6 [33.3]	1/7 [14.3]	14.5 ± 2.3	3/13 [23.1]
Wugni	1/2 [50.0]	0/3 [0.0]	13.8 ± 4.0	1/5 [20.0]
Total	21/100 [21.0]	20/106 [18.9]	15.5 ± 3.8	41/206 [19.9]

## Data Availability

Not applicable.
